# Special Economic Zone, Carbon Emissions and the Mechanism Role of Green Technology Vertical Spillover: Evidence from Chinese Cities

**DOI:** 10.3390/ijerph191811535

**Published:** 2022-09-13

**Authors:** Jieping Chen, Xianpeng Long, Shanlang Lin

**Affiliations:** School of Economics and Management, Tongji University, Shanghai 200092, China

**Keywords:** special economic zones (SEZs), green technology, vertical spillovers, carbon emissions, localized industry chain, multi-region input–output (MRIO) table

## Abstract

Although the special economic zones (SEZs) are considered the backbone of rapid economic development in China, it is unclear whether they contribute to green economic development. From the perspective of the localized industrial chains formed as a result of the SEZ policy, this paper aims to analyze and explain how the development of SEZs influences carbon emissions in Chinese cities by promoting green technologies’ vertical spillover along the industrial chain. Based on the panel data of 264 prefecture-level cities from 2011 to 2016 and a relatively new and mostly disaggregated city-level MRIO (multi-region input–output) table in China, this paper constructs green technology vertical spillover as a mechanism variable and discusses the influence theoretically and empirically. The results show that the development of SEZs can reduce a city’s carbon emissions. More specifically, for every 10 m^2^ increase in the size of the SEZ area, the carbon dioxide emission can be reduced by 0.882 g per m^2^ of the city area. Moreover, mechanism analysis shows that the development of SEZs promotes green technology vertical spillover inside the city, through which the SEZs reduce the city’s carbon emissions. The mediation effect occupies 21.96% of the total effect. Furthermore, the impact of the development of SEZs on carbon emissions has regional heterogeneity due to the city’s industry structure, green technology stocks, and the zones’ administrative hierarchies. The finding of this study could provide several important implications for regional green development, especially in China.

## 1. Introduction

Special economic zones (SEZs) have become a popular policy tool in many countries to promote economic development [1,2]. SEZs often have different names in different countries with various forms [3]. A general definition of a SEZ is a geographic area within a country that promotes certain economic activities through a range of policy tools that are generally not applicable to the rest of the country [4]. They are usually special zones and regions where the governments provide particular preferential policies and a favorable investment environment to attract enterprises to invest and settle down. However, they are not always uniformly successful, especially in Africa [5]. In China, the SEZs policy has been recognized as one of the most successful economic development policies [6,7,8,9]. To meet specific needs, China has implemented different forms of SEZs, including Economic and Technological Development Zones (ETDZs), simple “Development Zones (Kai Fa Qu)” at both national and provincial levels, Free Trade Zones (FTZs), Export-Processing Zones (EPZs), High-tech Industrial Development Zones, etc. [10]. The SEZs in China have contributed greatly to local economic growth and have become leaders in driving regional economic development. For example, in 2021, there were 230 national-level ETDZs throughout the country, accounting for about 11.98% of China’s total GDP. In the same year, the country’s 169 national high-tech zones create about 13% of the national GDP. Nevertheless, China’s current economic growth has been questioned as a traditional extensive economy with large energy consumption and pollution emission [11]. With China’s increasing emphasis on a green economy nowadays, can SEZs continue contributing to green economic growth, specifically by reducing carbon emissions? This is the main research question of this paper.

Although an extensive amount of the literature has discussed the relationship between industrial agglomeration and environmental quality, such as through carbon emissions [12], studies focusing on the influence of SEZs as industrial agglomerations within a city on carbon emissions are limited. Moreover, most are case studies or data analyses for one specific economic development zone [13,14]. Considering the localized industrial agglomeration or industrial chain brought about by the development of SEZs, this paper aims to investigate whether and how SEZs can influence regional carbon emissions in Chinese cities.

Unlike general industrial agglomerations, the formation of the SEZs results from a typical location-based industrial policy. At the beginning of the establishment of the zones, the government’s main planning is generally to determine the zone’s leading industries and target enterprises. In the subsequent period of investment attraction, the governments take the supporting and associated enterprises along the industry chain as the target enterprises in the zone. Industry-chain-oriented investment is the primary goal of the SEZs. As a result, the SEZs policy leads to the clustering of various enterprises from different industries along the industry chain, thus forming an industrial agglomeration in a city. Therefore, the development of SEZs is accompanied by stronger local upstream and downstream industrial linkages. This also promotes the spillover effect arising from buyer–seller links along the industry chain [15].

Many studies have focused on technological innovation as a major factor in reducing carbon emissions while neglecting green technology, which is more closely related to carbon emissions [16]. In recent years, scholars have also started to discuss the environmental impacts of green technology spillovers, such as carbon emissions [17]. Although there is a consensus that green technology spillover contributes to reducing carbon emissions [18], existing studies are insufficient in defining green technology spillover. Especially considering that global value chains (GVCs) have become localized and regionalized, e.g., in China, previous studies on foreign direct investment (FDI)-induced green technology spillovers or national or industrial level technology spillovers can no longer fully describe how green technology spillover affects carbon emissions. A study from Jiao and others (2020) [17] points out that the green technology vertical spillover resulting from the cooperation of upstream and downstream enterprises along the industry chain is conducive to creating a green, low-carbon, and environmentally friendly industry chain, thus reducing carbon emissions [17].

The Chinese government has recognized the importance of SEZs for green industry chains. The “Guiding opinions on accelerating the establishment of a sound economic system of green and low-carbon circular development” issued by China’s State Council in 2022 proposed two major initiatives to enhance the circulation level of economic zones and industrial clusters and to build green industry chains. This implies that the externalities of green technology spillover (GTS) from the industrial agglomerations in SEZs can help reduce carbon emissions. Many scholars have introduced mediating or mechanism variables into the discussion of environmental performance [19]. In this paper, green technology vertical spillover acts as a mechanism in the relationship between the development of SEZs and carbon emissions. To the best of our knowledge, no research has been done to include the development of SEZs, green technology vertical spillover, and regional carbon emissions into one unified explanatory framework. To fill the research gap, this paper pays special attention to the current localization and regionalization of industrial chains in China and, thus, the vertical spillover of green technologies along localized industrial chains induced by the Chinese SEZs policy. This paper investigates whether and how the economic zones can influence regional carbon emissions through green technology vertical spillover. More specifically, this paper presents two research questions. One is whether the development of SEZs can reduce regional carbon emissions. The other is to elaborate theoretically and empirically on the vertical spillover of green technologies as a mechanism through which the SEZs influence carbon emissions.

This paper contributes to the existing literature in the following ways. Firstly, it focuses on the industrial agglomeration and localized industrial chains induced by SEZ policies. It thus takes the vertical spillover along the chain as a mechanism through which the development zones can influence carbon emissions. Although a few studies have discussed the impact of economic zones on carbon emissions, the concept of green technology vertical spillover as a mechanism has not been incorporated into the theoretical framework so far. This study thereby provides a theoretical complement to the literature about green economic growth, the study of industry chains, and technology spillover. Moreover, previous literature has evaluated the indicator of vertical spillover through various methods and data. This paper constructs the indicator through a MRIO table which covers more subdivided sectors among all prefecture-level cities in China. The econometric measurement method and the data here are relatively new and comprehensive, making our results more accurate and reliable. In addition, the MRIO data also ensures that our empirical analysis is carried out on 264 prefecture-level cities from 2011 to 2016 in China. It advances previous related studies, mostly case studies or at the provincial level. Therefore, our results can better reflect the regional heterogeneity and provide practical significance for policy making.

The rest of this paper is organized as follows: Section 2 reviews the relevant literature. Section 3 introduces the background of SEZ policy in China and proposes the hypotheses. Section 4 describes the research design, including the econometric model and data source. Section 5 presents the empirical analysis and results. The last section concludes the paper.

## 2. Literature Review

This study is based on two main branches of literature. One is about the effect of the SEZs on environmental issues. The other is in the research fields most concerned with technology spillover, especially vertical spillover along the industry chain caused by the SEZ policies.

With the spread of the SEZs policy in China, research on the policy effects has received extensive attention from scholars. Literature has demonstrated the significant role of zones in enhancing regional economies, expanding exports, upgrading industrial structures, etc. [6,7,8,9,20]. Most studies agree on the role of zones in economic performance. However, controversy exists regarding their environmental effects. Some studies have found that SEZs cause spatial agglomeration of polluting firms and populations, which triggers an increase in industrial pollution and greenhouse gases emission, and thus exacerbates the environmental burden [21,22]. For example, Wang and Nie [23] found that the water quality of surrounding rivers deteriorated significantly in the short term after establishing the zones. In contrast, some researchers suggest that the development of special industry zones induces effects such as the monitoring-and-inspection effect, the economy-of-scale effect from public pollution treatment facilities, and the technological innovation promotion effect, which can ultimately improve the environmental performance of the region [24]. For example, Mohiuddin and others [10] revealed that China’s special zones serve as green economy incubators and agents for sustainable economic development. A study from Liu and others [25] takes the Beijing Economic and Technological Development Zone as a case study and argued that the greenhouse gas emission intensity of the zone decreased with the promotion of eco-industry. Similarly, research on special economic zones in Poland finds that cities with SEZs have lower PM emissions, and SEZs benefit the sustainable urban development in Poland [26].

While literature supports the positive effects of SEZs on the environment, most lack a generalized discussion of the relationship between the two at the city level. Moreover, regarding the mechanism analysis, no studies have yet paid attention to green technology vertical spillover along the localized supply chain caused by the zones. However, the effect technology spillover has on environmental issues has been recognized in research [27]. For example, Ilkay and others [28] investigated how the environment reacts to the development of globalization and human capital accumulation for countries in the BRICS group (Brazil, Russia, India, China, and South Africa). Their empirical results provide evidence for technology spillover acting as the mechanism through which globalization and human capital accumulation serve a sustainable environment. A number of studies exist that focus specifically on China. The study by Zhang and others (2021) explores how the technology spillovers brought about by import trade can indirectly reduce air pollution emission intensity in China. Similarly, Hao and others (2021) found that international technology spillover can reduce China’s carbon emissions as the regional intellectual property protection (IPP) level exceeds a threshold. Researches have demonstrated the existence of vertical technology spillovers and verified the positive effects of vertical technology spillovers on firm productivity, technological innovation, etc. [29,30,31]. For example, Le and Pomfret [32] found that vertical technology spillovers formed by FDI through upstream and downstream relationships in the industrial chain significantly increase domestic firms’ productivity by using Vietnamese firms’ data.

With the growing environmental problems, some studies have started to combine green technology with vertical technology spillover to explore the role of green technology in effectively balancing economic development and environmental protection [33,34,35]. Green technology vertical spillover is the phenomenon of green technologies spreading from one industry to another through industrial linkages [17]. Green technologies are embedded in intermediate products. They can be diffused directly to the downstream industries when the upstream industries provide products to the downstream ones along the supply chain or industry chain. In addition, different types of FDI can result in green technology spillover under different environmental regulation intensities [36]. Using panel data for the Italian region during the period 2002 to 2005, Ghisetti and Quatraro [37] found a significant positive impact from green technology innovation on corporate environmental protection in both intra-industry and vertically related sectors, demonstrating that vertical spillovers of green technology within related upstream and downstream industries drive improved environmental performance. Similarly, the study from Costantini and others [38] used industry-level data for 27 EU (European Union) countries from 1995 to 2009. It showed that green technologies developed in upstream sectors contribute to environmental performance at the national and international levels. In addition, the study reflected that green technology spillover among upstream and downstream components of the industrial chain is an important mechanism for the transition to a low-carbon, sustainable economy. This is in line with our view that the product transfer between upstream and downstream industries causes vertical spillover of green technologies.

Regarding the Chinese empirical setting, Jiao and others [17] applied industry-level input–output tables and panel data for 17 industries in China from 2000–2015 to analyze the relationship between green technology vertical spillover between industries and carbon emissions. They found a significant impact from green technology vertical spillover on reducing carbon intensity. Some other studies focus on the effects of vertical spillover of green technologies from a global industrial chain perspective [39,40,41]. These studies have acknowledged technology spillovers caused by upstream and downstream relationships along the industry chain or value chain. In particular, the study by Jiao and others [17] used input–output tables to construct a spillover indicator. However, their study only considered the economic linkages among 17 categories of industries in China. Differently, this paper applies the latest input–output tables developed by Chen and others [42], which is more segmented in spatial and industrial dimensions. As a result, the indicators we measure can better reflect the economic linkages between different regions in China.

Although we have recognized that the localized industrial chains formed by SEZs can cause vertical spillovers of green technologies, current studies on SEZs are mainly from the perspective of foreign direct investment, technology transfer, firm productivity, and labor markets [43,44,45]. A report from the World Bank shows that the government and administration of economic zones helps local enterprises connect with economic zone investors through supply chains for the sustainable development of SEZs [2]. The governments in China and South Korea encourages backward linkages in industry chains through technical assistance and other policy interventions [46]. The development of SEZs leads to the localization of industrial chains inside a city, allowing companies in different positions of the chain to form agglomerations or industrial clusters. Such linkages along the industry chain not only help achieve scale economies and business efficiencies, but also promote collaborative learning and improve competitiveness [47]. Meanwhile, the closer upstream and downstream linkage of industry chains induced by SEZs implies a higher degree of vertical spillover of technology.

Altogether, the literature review shows the existence of a positive effect of green technology vertical spillover on environmental performance. However, very few studies pay attention to the vertical green technology spillover issue caused by the SEZs. To address the research gap, this study discusses whether the development of SEZs can affect the level of carbon emissions in cities through vertical spillover of green technologies.

## 3. Policy Background and Hypotheses

Although the SEZ policy has been in China for a very long time, it was not until 1999 that the zones began to enter a phase of rapid development. During this period, the government formulated and implemented policies to guide the development of the zones toward industrial clustering. In 1999, the Ministry of Science and Technology issued the “Opinions on Accelerating the Development of National High-tech Industrial Development Zones”, which proposes that the zones follow the investment model based on industrial chains for promoting the development of industrial clusters. Since then, investment attraction focusing on industrial chains has become the primary development mode of the SEZs. The development of the zones has shifted from simple scale expansion to a new stage of growth driven by the leading industries and supporting industrial chain extension.

After that, with the background of international technology transfer at the beginning of the 21st century, the SEZ policies began to emphasize the introduction, absorption, and reinvention of technology, such as in the “Circular on Several Opinions on Promoting the Further Development of National High-tech Industrial Development Zones to Enhance Independent Innovation Capability” jointly issued by the National Development and Reform Commission, the Ministry of Science and Technology, and the Ministry of State-owned Assets in 2007. Since 2009, the government’s policy guidance for SEZs has focused on innovation, coordination, being green, openness, and sharing, with a particular emphasis on building green parks. Subsequently, the China State Council launched “Opinions on Promoting the Transition, Upgrading and Innovative Development of National Economic and Technology Development Zones” and “Opinions on Promoting the Reform and Innovative Development of Development Zones” in 2014 and 2017, respectively [48]. These policies emphasize the need to encourage enterprises’ cooperation and innovation, extend the industrial chain, and enhance the agglomeration effect. Therefore, the SEZs are developing into comprehensive parks by attracting investment based on industrial chains, which has always been their main development model.

In fact, attracting enterprises based on industrial chains is the primary mechanism for SEZs to realize localized industrial chains, form closer upstream and downstream industrial linkages, and further generate vertical technology spillovers. Following industrial investment, SEZs can fully play into the agglomeration effect of industry-chain-related enterprises and form industrial clusters. A study by Moberg [49] finds that the government sets rules in some special economic zones for the type of products, industries, and export performance. These rules and requirements make the zones resemble national-planned industrial clusters. Moreover, the global industrial chain has entered a period of reformation and transformation, showing new characteristics of localization and regionalization. In the case of China, the localized industrial chain can reduce risks and maintain stability in the economy. Currently, many local governments in China have issued guiding policy documents to emphasize industrial chain support in SEZs. Benefiting such an industrial-chain-supporting development model, the development of SEZs is accompanied by a higher degree of upstream and downstream industrial chain linkages.

This higher degree of upstream and downstream linkage along the industry chain within the SEZs can thus form a green technology vertical spillover between upstream and downstream industries [50,51]. On the one hand, upstream enterprises realize green technology vertical spillover by providing downstream enterprises with intermediate products embedded with advanced green technology. On the other hand, the technological innovation of downstream enterprises triggers demand-pull for higher technical requirements on intermediate products, thus stimulating upstream enterprises to upgrade green technology, thus realizing vertical spillover of green technology between upstream and downstream industries in the industrial chain [52].

In summary, both the investment-attraction mode based on the industrial chain and industrial-chain-supporting policies for SEZs can promote stronger upstream and downstream linkages along the industrial chain, leading to green technology vertical spillover. As a result, we put forward the following hypothesis of this paper.

**Hypothesis** **1.** *The development of SEZs can effectively promote green technology vertical spillover.*

Literature has shown that green technology is a critical way to effectively alleviate the dual pressures of energy and the environment [33,35,53]. Meanwhile, green technology spillovers and their role in improving environmental performance have also been testified to in research [41,54,55]. For example, the study by Jiao and others [17] proves that the vertical spillover of green technology can significantly reduce the carbon emission intensity in China. Policymakers are also aware of the significance of the local industry chain formed in economic development zones for carbon emissions. In 2016, the State Council of China issued the “Notice on the Work Plan for Controlling Greenhouse Gas Emissions,” which clearly puts forward measures to promote cooperation between upstream and downstream enterprises, create a green, low-carbon, and environmentally friendly industrial chain, and reduce carbon emissions.

Firstly, the vertical spillover of green technology encourages enterprises to produce green technology innovations, which directly reduce carbon emissions in the production process. Moreover, through the connection between upstream and downstream components of the industry chain, upstream enterprises provide downstream enterprises with intermediate products embedded with advanced green technology, thus indirectly reducing the carbon emissions in the production process of downstream enterprises. Furthermore, the interconnection between upstream and downstream forms a cyclic cumulative effect. Specifically, transactions between upstream and downstream result in knowledge and technology spillover due to frequent interactions, further promoting the green technology innovation capabilities of the participants. As a result, the carbon emissions within the whole region are reduced due to the improved green technology and the vertical spillover effect. Therefore, we propose the second hypothesis of this paper.

**Hypothesis** **2.** *The development of SEZs can reduce carbon emissions by increasing the vertical spillover of green technologies in the cities where they are located.*

## 4. Research Design

**Figure 1:**
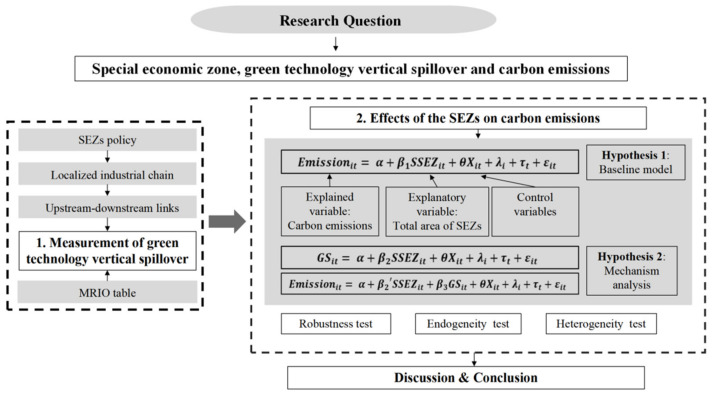
Research framework (Source: Own creation).

Regarding the research questions of this paper and the two proposed hypotheses, we need to quantify the green technology vertical spillover first and then test the hypotheses and the mechanism. Figure 1 shows the research framework of this paper.

### 4.1. Econometric Model

As stated above, many studies on the determinants of environmental performance have introduced discussion of mechanism variables. Various econometric methods and software packages are used for empirical verification. For example, Del Brío and Barba-Sánchez [19] apply structural equation modeling (SEM) to discuss the mediating effect of environmental proactivity on the influence of stakeholder pressure and environmental strategy with a sample of 312 Spanish wineries. Unlike their cross-sectional data sample, our research is based on panel data. Generally speaking, the pooled OLS (Ordinary Least Squares) model, fixed effect model, and random effect model can be used for panel data as econometric methods. Because we need to observe the changes in each city in the time series, the pooled OLS model is not applicable in this paper. We then construct the Hausman test for the selection between fixed or random effect models. In the Hausman test, the chi^2^ statistic is 364.19 with *p*-value < 0.001. Therefore, the hypothesis that explanatory variables are unrelated to individual effects is rejected. Consequently, this paper applies the two-way fixed effect model to empirically study the impact of the development of the SEZs on carbon emissions. The benchmark model is set as follows:

(1) $$Emission_{it}=\alpha +\beta_{1}SSEZ_{it}+\theta X_{it}+\lambda_{i}+\tau_{t}+\varepsilon_{it}$$

where $Emission_{it}$ represents the carbon emission per unit area of the city i in year t; $SSEZ_{it}$ is the total area of the zones in the city i in year t; $X_{it}$ is a set of city-level control variables; $\lambda_{i}$ is city-fixed effect and $\tau_{t}$ is year-fixed effect; $\varepsilon_{it}$ is the random error term; α is a constant term; and both $\beta_{1}$ and θ are estimation coefficients. Among them, $\beta_{1}$ is the parameter of concern in this paper.

Furthermore, we need to identify the mechanism effect through which the zones influence a city’s carbon emissions. Referring to the study by Persico and others [56], we have:

(2) $$GS_{it}=\alpha +\beta_{2}SSEZ_{it}+\theta X_{it}+\lambda_{i}+\tau_{t}+\varepsilon_{it}$$

(3) $$Emission_{it}=\alpha +\beta_{2}{}^{\prime }SSEZ_{it}+\beta_{3}GS_{it}+\theta X_{it}+\lambda_{i}+\tau_{t}+\varepsilon_{it}$$

where $GS_{it}$ represents the green technology vertical spillover. $\beta_{2},\beta_{2}\prime$, $\beta_{3}$ is the parameter we are concerned with in this paper. If $\beta_{2}$ > 0 in Model (2), the size of the zones significantly improves the green technology vertical spillover. If $\beta_{2}$ > 0, *GS* is added to Model (3) to test further the impact mechanism of the zones on carbon emission.

### 4.2. Variable Construction

(1) *Explained variable*

This paper uses the carbon dioxide emission per unit area of the city as the indicator for the carbon emission in a city. We refer to the method from Ren and others [57]. It measures the city-level carbon dioxide emission through three types of energy consumption: natural gas, liquefied petroleum gas (LPG), and electricity. The measurement equation is as follows:

(4) $$CO_{2}=kE_{1}+vE_{2}+\varphi \left(\eta \times E_{3}\right)$$

where $E_{1}$ is the consumption amount of natural gas; k is the $CO_{2}$ conversion coefficient of natural gas, which is 3.1013 kg/(kW·h); $E_{2}$ is the consumption amount of LPG; v is the $CO_{2}$ conversion coefficient of LPG, which is 2.1622 kg/(kW·h); $E_{3}$ is the consumption amount of electricity; η is the proportion of coal power in total electricity or power generation; and φ is the greenhouse gas emission coefficient of coal power fuel chain, which is 1.3203 kg/(kW·h).

(2) *Explanatory variable*

This paper uses the total area of zones ($SSEZ_{it}$) as the indicator for the development of special zones in a city. Unlike the indicator of the existence of zones in a city, the total area reflects the possibility of clustering of different enterprises along the industry chain. Meanwhile, it represents the scale of the spatial agglomeration in a city to a certain extent.

(3) *Mechanism variable: green technology vertical spillover*

To measure the green technology vertical spillover in a city, we need to have the stock of green technologies and the spillover weight inter-industry within a city.

Firstly, we calculate the stock of green technologies for each industry in each city.

Step (1), referring to the study from Hu and others [58], we use green technology patents to measure green technology innovation. According to the list “IPC (international patent classification) Green Inventory” published by the WIPO (World Intellectual Property Organization) in 2010, we identify the patent-application records of green technologies during the period 2000–2017 from the Intellectual Property Patent Database of the State Intellectual Property Office of China (SIPO). There are three types of patents in China: invention, utility model, and external-design. Considering the relatively low level of innovation-degree in external-design patents, this paper counts the first two patent types, namely, invention and utility model. As a result, we retrieve 2,813,962 valid green technology patents out of 26,783,987 patent application records.

Step (2), according to the “International Patent Classification and National Industry Classification Reference Relationship Table (2018)” issued by the SIPO, we categorize the green patent application records by year, city, and industry through the classification number and address information of each record. Thus, we obtain the number of green patent applications for 42 industries in 313 cities from 2000 to 2017.

Step (3), we refer to the study by Jiao and others [17] and adopt the perpetual inventory method by Braun and others [59] to calculate the green technology patent stock as follows:

(5) $$GT_{i,k,t}=PAT_{i,k,t}+\left(1-\delta \right)GT_{i,k,t-1}$$

(6) $$GT_{i,k,0}=\frac{PAT_{i,k,0}}{g+\delta }$$

where $GT_{i,k,t}$ is the green technology stock of industry *k* in city *i* in year *t*; δ is the depreciation rate and is set to 20% according to Braun and others [59]; g is the average growth rate of green patent technology applications from 2000 to 2017, and is equal to 28.08%; $PAT_{i,k,t}$ is the number of green technologies in industry *k* in city *i* and year *t*; and $GT_{i,k,0}$ is the green technology stock in the first year. Using Equations (5) and (6), we can have the respective green technology stocks of 42 industries in 313 cities between 2000 and 2017.

Secondly, we refer to the study by Jiao and others [17] to calculate the vertical technology spillover weights.

Literature has widely acknowledged the technology embodied in intermediates and the technology spillover along the flow of intermediate deliveries between upstream and downstream sectors in an input–output matrix [60]. Estimates of such spillover are traditionally based on the weighted technical or scale coefficients of production of an input–output matrix [61]. Therefore, this paper uses the proportion of the direct consumption coefficient of intermediate goods in the input–output table as the spillover weights among different industries. More specifically, we can obtain the inter-sectoral or vertical spillover from the upstream to the downstream sector by multiplying the green technology stock of the upstream sector with the proportion of its direct consumption of intermediate products in the downstream sector. The equation is as follows:

(7) $$GS_{i,k}=\underset{j}{\sum }\left(\frac{I_{k,j,i}^{}}{\sum_{i}\sum_{j}I_{k,j,i}^{}}\times GT_{i,j}\right),\;\;\forall j\neq k$$

where $I_{k,j,i}^{}$ is the direct consumption coefficient of intermediate goods produced by industry j in city i to industry k in the input–output table; $\frac{I_{k,j,i}^{}}{\sum_{i}\sum_{j}I_{k,j,i}^{}}$ is the ratio of the consumption of the intermediate goods from industry *k* to industry *j* to the all-intermediate-goods consumption from all industries in all cities to industry *j*; and $GT_{i,j}$ is the green technology stock of industry j of the city i.

Finally, we get the total green technology vertical spillover for the city i, $GS_{i}$, as follows:

(8) $$GS_{i}=\frac{1}{100}\sum GS_{i,k}$$

(4) *Control variables*

To reduce the estimation error induced by omitted variables, this paper applies six control variables, including the GDP per capita (*gdp*) as an indicator for regional economic growth, the ratio of secondary industry output to GDP (*structure*) to indicate the industrial structure in a city, the governmental scientific expenditure per capita (*tech_exp*), the population of a city (*population*), the annual retail sales of consumer goods per capita (*consumption*) to represent consumption capacity, and the actual amount of foreign capital used (*open*) to indicate the degree of openness.

### 4.3. Data Description

**Figure 2:**
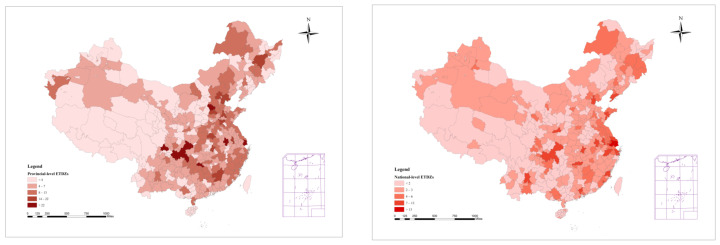
Distribution of two types of ETDZs in 264 prefecture-level cities in China (**left**: provincial-level zones; **right**: national-level zones). Source: Own creation, results of ArcGis (Esri, Redlands, CA, USA).

The dataset in this paper is mainly from the China City Statistical Yearbook coedited by NBS (National Bureau of Statistics). The zone data comes from the “China Development Zone Review Announcement Catalogue (2018)” issued by the Chinese State Council. The catalogue provides information on all development zones approved and established by the Chinese State Council and provincial governments, including ID, name, province, approval time, approved area, leading industry, and other information. We obtain each zone’s latitude and longitude information through the geocoding service API provided by the Baidu Map open platform. The geocoding API can convert structured address data into corresponding coordinate points (latitude and longitude). And the service address is https://lbsyun.baidu.com/index.php?title=webapi/guide/webservice-geocoding (accessed on 1 May 2022). Then, by using the geographic inverse coding service API, we obtain the city name where each zone is located. After that, we have the total zone area for each city each year. Since 2010, the development of SEZs has undergone rapid expansion. Considering the data availability, we select the period from 2011 to 2016 for the empirical analysis. The research sample covers 264 prefecture-level cities in China and includes 535 national-level ETDZs and 1989 provincial-level ETDZs, 2524 zones altogether. Figure 2 shows the distribution of the two types of ETDZs in the 264 cities in China. The national-level development zones are approved by the State Council, while provincial-level development zones are approved by provincial, autonomous, and municipal governments. In addition, national-level zones have advantages over provincial-level ones in terms of size, strength, and access to preferential policies. The MRIO table used to measure green technology vertical spillover is from the study by Chen and others [42]. Compared to currently available Chinese MRIO tables, which have a very low resolution at the regional (or provincial) and sectoral levels, this table includes 297 prefecture-level cities and 28 sectors. It can directly reflect the trade relations and patterns among different industries in each region or city. Table 1 reports the descriptive statistics of the main variables.

## 5. Empirical Results and Analysis

### 5.1. Baseline Model

This paper uses Stata version 17 (StataCorp, College Station, TX, USA) to carry out all regression models. Table 2 reports the regression results of Model (1). Results show that the coefficient of the main explanatory variable $\left(SSEZ\right)$ is always significantly negative in all situations, controlling for time- and city-fixed effects. The coefficient of SSEZ of −0.882 in Column (7) indicates that for every 10 m^2^ increase in the size of the SEZ area, the carbon dioxide emission can be reduced by 0.882 g per m^2^ of the city area (the original measurement unit is in 1000 hectares for the size of the development zone and 10,000 tons/ha for the emissions). Therefore, the increase in the zones’ area can significantly reduce the carbon emissions in a city, which validates Hypothesis 1 in Section 3. In addition, the coefficients of two control variables (*structure*, *population*) are significantly positive, indicating that a city’s share of secondary industry and population density can significantly increase carbon emissions. Meanwhile, the coefficients of the variable (tech_exp) are significantly negative, showing that the scientific expenditure of cities can significantly reduce the carbon emission level. The other three variables (*gdp*, *open*, *consume*) have no significant effect on a city’s carbon emission.

To further test the effects of the SEZs on the city’s carbon emissions, we use a DID (Difference-in-Differences) approach with the existence of new SEZs as a policy variable. Because most cities had provincial-level SEZs from 2011 to 2016 (about 11.7% of cities have no SEZs), we use the existence of national-level SEZs as the main explanatory variable in Model (4).

The model is set up as follows:

(9) $$emi\_capita_{it}=\alpha +\beta_{1}^{\prime }D_{it}+\theta X_{it}+\lambda_{i}+\tau_{t}+\varepsilon_{it}$$

where $emi\_capita_{it}$ is the carbon emissions per capita in city i in year t; $D_{it}$ is the dummy variable, if the city i has a new national-level SEZ in year t, then $D_{it}=1$ for the year and the subsequent years, otherwise $D_{it}=0$; $X_{it}$ is a set of city-level control variables; $\lambda_{i}$ is city-fixed effect; $\tau_{t}$ is year-fixed effect; $\varepsilon_{it}$ is the random error term; α is a constant term; and both $\beta_{1}^{\prime }$ and θ are estimation coefficients.

Columns (1) and (2) of Table 3 show the regression results of Model (4) without and with control variables, respectively. The data in Column (2) indicate that the existence of SEZs can significantly reduce carbon emissions in cities, resulting in a reduction of 152,985 kg of carbon emissions per capita.

Furthermore, the parallel trend assumption is an important prerequisite for applying DID. It requires the existence of the same trend in the outcome variable of the observed sample with the absence of policy shocks. To ensure the stability and reliability of DID results, we conduct the parallel trend test for Model (4). The estimating equation is as follows:

(10) $$emi\_capita_{it}=\alpha +\overset{2}{\underset{n=-2}{\sum }}\rho_{n}D_{it}^{n=t-k}+\theta X_{it}+\lambda_{i}+\tau_{t}+\varepsilon_{it}$$

where $D_{it}^{n=t-k}$ is a dummy variable; *k* is the year of establishment of new SEZs in each city; and *n* is the standardized pre- and post-policy period, *n* < 0 for the pre-policy period, *n* = 0 for the current policy period, and *n* > 0 for the post-policy period. $D_{it}^{n=t-k}$ is taken as 1 if *t* − *k* is equal to *n* for a city, and 0 otherwise.

The results of the parallel trend test are reported in Column (3) of Table 3. The coefficients of the dummy variables are not significant in the periods before the establishment of the new SEZs. In contrast, the coefficients become significant following the year of the establishment of the new SEZ. This implies that the trend of carbon emission per capita is consistent before the policy shock. The hypothesis of the parallel trend is valid. After the policy shock, carbon emissions show a significant decreasing trend, which further indicates that the establishment of new SEZs negatively affects the city’s carbon emissions.

### 5.2. Mechanism Examination

Table 4 reports the results of the mechanism examination. Both coefficients of the SEZs affecting the mechanism variable (*GS*) in Columns (1) and (2) (without or with the control variable) are significantly positive at the 1% level, indicating that the increase in the total area of the zones can significantly increase the vertical spillover of green technology in cities. After the mechanism variable is added to the model in Column (3) and Column (4), respectively, the significance of the main explanatory variable remains unchanged compared to the baseline model in Table 2. In addition, the coefficient of the SEZs variable changes from −0.882 (in the baseline regression model, Column (7) of Table 2) to −0.718 (Column (4) with control variable). The amount of the direct effect of SEZs on carbon emission is -0.718, and that of its indirect effect, namely the mediation effect, is −0.1937 (=3.459 × (−0.056)), which occupies 21.96% (=3.459 × (−0.056)/(−0.882)) of the total effect. It means that the size of zones can reduce carbon emission by increasing the green technology vertical spillover of cities, thus verifying Hypothesis 2 proposed in this paper.

### 5.3. Robustness Check

To ensure the reliability of the regression results, this paper applies three robustness tests. (1) Exclude the sample of cities without new zones after 2011. In our sample, some cities were not approved to add new zones after 2011. Considering that our research period is 2011 to 2016, we exclude these cities. (2) Change the indicator for the main explanatory variable SSEZ. Here, we use the cumulative approved area of the zones from 2011 to year i to measure the variable SSEZ, instead of the total approved areas. (3) Replace the indicator for the mechanism variable. We use the MRIO table from the study by Zheng and others [62] to measure green technology vertical spillover instead of the table from the study by Chen and others [42]. The results in Table 5 remain consistent with those in baseline regression, showing that our models are robust.

### 5.4. Endogenous Issues

The carbon emissions of cities and the establishment of zones are influenced by many factors. In this paper, we add year- and city-fixed effects and related control variables in the Models (1)–(3) to solve the endogeneity problem caused by omitted variables to some extent. However, SEZ policy has been used by some local governments as a policy tool to create a green economy, such as through the establishment of green economic development zones and low-carbon industrial parks, etc. The local governments might establish green development zones because of carbon emission pressure. Therefore, our models might have an endogeneity problem due to reverse causality.

This paper adopts the instrumental variable (IV) approach to tackle the endogeneity problem. An ideal IV needs to satisfy two conditions: (1) it must be (highly) correlated with the endogenous variable; and (2) it is uncorrelated to the disturbance term.

Here we use the inverse of the relief-degree of the land surface (I_rdls) measured by You and others [63] as an instrumental variable for the endogenous variable SSEZ. On the one hand, the relief-degree of the land surface of a city is a natural geographical feature of the city and has no direct correlation with carbon emissions. On the other hand, economic zones are usually built on flat land that is easy to construct upon. The higher the land surface’s relief degree, the lower the possibility for the land to become an economic zone. Therefore, the IV (I_rdls) can meet both relevance and exogeneity requirements and is a suitable instrument for our analysis. Since the IV (I_rdls) is cross-sectional data, we refer to the method by Angrist and Keueger [64] and use the interaction term (I_rdls* year) in the empirical analysis. In addition, we refer to the study from Ghisetti and Quatraro [65] and use the first-order lag term ${\mathrm{GS}}_{t-1}$ as the IV for the endogenous variable GS.

This paper applies the two-stage least squares (2SLS) regression for the endogeneity test. The Equations (11) and (12) are the first stage of the 2SLS and Equations (13)–(15) are the second stage.

(11) $$SSEZ_{it}=\alpha +\beta_{1}I\_rdls+\theta X_{it}+\lambda_{i}+\tau_{t}+\varepsilon_{it}$$

(12) $$GS_{it}=\alpha +\beta_{1}GS_{t-1}+\theta X_{it}+\lambda_{i}+\tau_{t}+\varepsilon_{it}$$

(13) $$Emission_{it}=\alpha +\beta_{1}{}^{\prime \prime }prob\_SSEZ_{it}+\theta X_{it}+\lambda_{i}+\tau_{t}+\varepsilon_{it}$$

(14) $$GS_{it}=\alpha +\beta_{2}{}^{\prime \prime }prob\_SSEZ_{it}+\theta X_{it}+\lambda_{i}+\tau_{t}+\varepsilon_{it}$$

(15) $$Emission_{it}=\alpha +\beta_{2}{}^{\prime \prime }prob\_SSEZ_{it}+\beta_{3}{}^{\prime \prime }prob\_GS_{it}+\theta X_{it}+\lambda_{i}+\tau_{t}+\varepsilon_{it}$$

Table 6 reports the results. The F-statistics of both instrumental variables in Columns (1) and (2) are greater than 10. Therefore, the weak instrumental variables can be excluded. It is worth noting that the estimated coefficients of variables I_rdls_2011 and I_rdls_2012 on SSEZ are both significantly negative in Column (1). It is not consistent with our expectation that economic zones are usually built on land with lower relief degrees. A study from Sun and others [66] applies the urban slope index as an instrumental variable for the level of urban transportation infrastructure. They find that cities with a higher urban slope have better transportation infrastructure, while cities with better transportation infrastructure are more likely to have economic zones. Therefore, it is reasonable that the inverse of the relief-degree of the land surface (I_rdls) shows a significant negative correlation with the variable SSEZ, the size of the economic zone. In addition, the coefficient of variable SSEZ in Column (3) is significantly negative, indicating that the development of SEZs can significantly reduce carbon emissions after solving the endogeneity issues. The coefficient of variable SSEZ in Column (4) is significantly positive, indicating that the development of the zones significantly increases the green technology vertical spillover inside a city. Moreover, both coefficients of the two variables SSEZ and GS in Column (5) are significantly negative, and the absolute value of the coefficient for variable SSEZ is smaller than that in Column (3). Therefore, we can conclude that developing SEZs can significantly reduce carbon emissions through the green technology vertical spillover effect.

### 5.5. Heterogeneity Analysis

Considering that the heterogeneity of various cities might lead to the differences in the intensity of the influence of the SEZs on carbon emission, as well as the vertical spillover, this paper applies three indicators to carry out the heterogeneity analysis.

#### 5.5.1. Heterogeneity Analysis by Industry Structure

Generally speaking, primary and secondary sectors are more like to produce pollutant emissions than the tertiary sector. Moreover, for cities with a large tertiary sector, the development zones are more likely to attract enterprises focusing on technological innovation and productive services, which usually have a more significant effect on carbon emission reduction. This paper splits the whole sample by measuring the tertiary industry output ratio to each city’s GDP. Specifically, all samples are categorized into two groups (low-ratio group and high-ratio group) according to the median value of the ratio (37.3%). Results in Table 7 show that no significant relationship exists between the size of the zones and carbon emission in the low-ratio group. In contrast, in the high-ratio group, the development of the SEZs significantly reduces carbon emissions ($\beta_{1}$ = −1.323, *p* < 0.01). Moreover, it significantly promotes the vertical spillover of green technology ($\beta_{2}{}^{\prime }$ = −1.144, *p* < 0.01; $\beta_{3}$ = 4.404, *p* < 0.01), and the mechanism effect holds. The result verifies again that the control variable (*structure*) negatively influences carbon emissions in the baseline model.

#### 5.5.2. Heterogeneity Analysis by Green Technology Stock

As stated above, the intensity of vertical spillover relates to the green technology stock at the city and industry levels while the vertical spillover of green technology has a positive effect on reducing carbon emission. In addition, green technology, as an intangible asset, can lead to knowledge-sharing externalities. The agglomeration of green technologies inside a city can be an influential factor for enterprises’ choice of SEZs. Therefore, we categorize the whole sample into two groups by measuring the green technology stock variable. Specifically, the samples are classified into the low and high groups based on the median value (0.5583). The results are in Table 8. The coefficients of SSEZ in Column (3) are not significant in the group with low green assets. In contrast, significant effects are observed in all three columns (Columns (4)–(6)) in the group with higher green technology assets ($\beta_{1}$ = −1.187, *p* < 0.01; $\beta_{2}{}^{\prime }$ = −1.038, *p* < 0.01; $\beta_{3}$ = 3.812, *p* < 0.01). This suggests that cities with a high level of green technology can significantly reduce carbon emissions through the spillover effect resulting from the localized industry chain.

#### 5.5.3. Heterogeneity Analysis by Administration Hierarchy of SEZs

In our observed sample, two types of zones are included, the national- and the provincial-level zones. Due to the different administrative levels, these two types of zones enjoy different preferential policies. Moreover, the national-level zones are usually located in central cities such as provincial capitals, open-coastal cities, and other open cities. These cities have better infrastructure and industrial bases, more sufficient human resources, and a larger potential market scale than other cities. Meanwhile, they have higher land and human capital costs and greater market competition than others. Therefore, we categorize all zones into two groups according to their administrative level.

The results in Table 9 show that significant effects exist in all models for national zones ($\beta_{1}$ = −1.904, *p* < 0.01; $\beta_{2}{}^{\prime }$ = −1.589, *p* < 0.01; $\beta_{3}$ = 7.057, *p* < 0.01), while there is no significant relationship in the provincial zones. Due to their policies and geographical location, the national zones can attract companies with more advanced green technologies and higher levels of innovation. As a result, they have a more significant effect on green technology spillover and carbon emissions.

## 6. Discussions

To address the research questions, this paper presents two hypotheses through theoretical analysis. One hypothesis is that the development of SEZs can effectively promote green technology vertical spillover. The other is that the development of SEZs can reduce carbon emissions by increasing the vertical spillover of green technologies in the cities where they are located. That means the green technology vertical spillover works as a mechanism through which the SEZs reduce the carbon emission of a city. To test the hypotheses, this paper applies city-level panel data covering 264 prefecture-level cities from 2011 to 2016 in China to ensure reliable empirical results. In addition, to measure the technological vertical spillover, we employ an advanced MRIO table [42], which covers 297 prefecture-level cities and 28 sectors in China.

According to the results of the baseline model, increase in the zones’ area can significantly reduce the carbon emission in a city (Hypothesis 1: $\beta_{1}$ = −0.882, *p* < 0.05), validating the first hypothesis; specifically, for every 10 m^2^ increase in the size of the SEZ area, the carbon dioxide emission can be reduced by 0.882 g per m^2^ of the city area. While SEZ policy has led to success or failure in different countries [5], its positive contribution to Chinese economic development is no longer in doubt. However, regarding the green economy or environmental performance in China, its effect is still controversial. The findings of previous studies are mixed. As mentioned earlier, some studies have concluded that the development of SEZs has had a damaging impact on the environment [23]. Still, others treat it as beneficial [10]. Because most of this research is case studies or in different empirical settings, the findings lack general applicability. Firstly, most of the existing studies about SEZs adopt a case study approach [3] while different kinds of SEZs have differences in administrative levels, resources, and privileges, all of which can lead to diverse performance levels and thus various research findings. For example, Shi and others [13] use an ETDZ in Tianjin, China as a case study and found that the zone can have a series of environmental benefits. Their conclusion may also be biased because the development zone itself is positioned as an ecological industrial park. Secondly, many studies on SEZs’ environmental effects have been conducted over various periods in different countries or regions. For example, the Chinese government has been emphasizing the construction of green parks since 2009, making the development of green and ecological parks one of the main development goals in the future. Therefore, an empirical analysis using the period after 2009 in China may be able to draw some conclusions in favor of the environment.

The second hypothesis about the mechanism examination of green technology vertical spillover has also been tested (Hypothesis 2: $\beta_{2}$ = 3.459, *p* < 0.01; $\beta_{3}$ = −0.718, *p* < 0.05). The mediation effect of the variable (*GS*) as an indirect effect occupies 21.96% of the total effect. It means that the development of SEZs can significantly increase the green technology vertical spillover within cities, which ultimately reduces a city’s carbon emissions. The result about the relationship between technology spillover and carbon emission is generally consistent with those of previous studies, such as Yang and others [27] and Hao and others [67]. Previous literature has also studied the mechanism effect of the technology spillover and discussed its positive impact on environmental issues [68]. As mentioned above, the study from Jiao and others [17] discusses the indirect role of green technology vertical spillovers on carbon intensity. We refer to their methodology for measuring the green technology vertical spillovers between green industries. Our data include almost all prefectural-level cities (264 cities) and 28 sectors in China, while the study from Jiao and others [17] uses the panel data of 17 categories of industries in China. The measurement of green technology vertical spillover is thus one of the highlights of this paper.

In addition to the two hypotheses, this paper conducts a heterogeneity analysis, showing the existence of diversification in cities and zones. More specifically, the significance and magnitude of the influence are prominent in areas with a high ratio of tertiary industry ($\beta_{1}$ = −1.323, *p* < 0.01; $\beta_{2}{}^{\prime }$ = −1.144, *p* < 0.01; $\beta_{3}$ = 4.404, *p* < 0.01) or cities with more green technology stock ($\beta_{1}$ = −1.187, *p* < 0.01; $\beta_{2}{}^{\prime }$ = −1.038, *p* < 0.01; $\beta_{3}$ = 3.812, *p* < 0.01). Also, the national zones exhibit a significant effect in promoting green technology vertical spillover and thus reducing carbon emissions ($\beta_{1}$ = −1.904, *p* < 0.01; $\beta_{2}{}^{\prime }$ = −1.589, *p* < 0.01; $\beta_{3}$ = 7.057, *p* < 0.01). All results reflect that the green technology vertical spillover is the main mechanism through which a city’s development zones reduce the carbon emission inside the city.

## 7. Conclusions 

The SEZ policy has contributed to China’s high rate of economic growth. In the face of today’s demand for sustainable growth, can the current development of SEZs contribute to green economic growth? Based on this inquiry, this paper examines the impact of SEZs on regional carbon emissions. Moreover, from the perspective of the localized industrial chains formed as a result of the SEZ policies, this paper investigates how the development of SEZs can ultimately affect carbon emissions by promoting green technologies vertical spillover along the industrial chain. 

Theoretically, this paper contributes to the literature about green economic growth, the study of industrial agglomeration, and input–output research. While the factors affecting carbon emissions have been widely studied, little attention has been paid in the literature to the influence of SEZs policies. In addition, we identify the vertical spillover effect resulting from the development of SEZs, which complements theoretically and empirically the agglomeration and technology-spillover research. Furthermore, this paper adopts a MRIO table to measure the vertical spillover. To the best of our knowledge, no prior analysis has been carried out on almost all prefecture-level cities in China. This is one of the newest insights of this study.

The findings of this study could provide several important implications for regional and national policymakers, especially in China. Firstly, the main result regarding the positive effect of SEZs on carbon emissions shows the value of current policies to encourage green economic development in SEZs. In the process of building economic development zones, we should pay attention to the inter-industry relationship of different enterprises, enhance the formation of industrial parks and industrial clusters, and improve the industrial chain to promote technological spillover between upstream and downstream industries. The development of SEZs needs to balance industrial linkages inside the zones, and avoid homogeneous and low-technological industries. Secondly, the findings of the mechanism analysis indicate that green technology vertical spillover has a significant contribution to reducing carbon emissions. This conclusion has been demonstrated in the previous literature [27]. This reinforces the importance of emphasizing green technology and green innovation for the sustainable development of the economy. Therefore, it is necessary both to encourage cross-regional and cross-enterprise cooperation and to increase the overall enthusiasm for innovation [69]. Thirdly, our research demonstrates the positive effect that industrial agglomeration caused by development zones has on green technology spillovers. This shows that the optimization of the industrial structure due to rational agglomeration can facilitate knowledge spillovers as well as spillovers caused by product-transmission relationships between up- and downstream institutions along the industrial chain. Therefore, it is more important for governments at all levels to utilize SEZ policies for rational resource allocation and industrial transformation. Finally, the heterogeneity analysis shows the difference in the intensity of the influence. Although the development of SEZs is beneficial to economic and environmental development, duplication is unnecessary. The establishment and expansion of SEZs should focus on regional endowments and location advantages. The management department of economic zones should advocate regional green development and make development zones a vital force in the high-quality development of the regional economy.

Nevertheless, this study suffers several limitations. As stated above, a variety of different SEZ concepts exist in China. Due to data availability, this paper applies the zone data from the “China Development Zone Review Announcement Catalogue” of 2018 which mainly contains information on the ETDZs. Different forms of SEZs may have various impacts on carbon emissions due to their respective policies and development priorities. A more specific categorization of SEZs should make our findings more reliable. In addition, concerns might be raised about the applicability of the data. The relatively short period (from 2011 to 2016) examined is a period of economic conjuncture. Therefore, the results may be tendentious for further application. We have applied different econometric methods to make our results more reliable. A longer period could be chosen for future studies. Furthermore, environmental burden such as carbon emissions depends on economic levels, technological advancement, and institutional settings. Future research can account for institutional-related indicators and may yield more relevant progress.

## Figures and Tables

**Table 1:** Descriptive statistics and data sources.

	(1)	(2)	(3)	(4)	(5)	(6)
Variables	*n*	Max	Min	Mean	Sd	Data Sources
*Emission*	1584	357.5	0.731	32.95	36.91	China Energy Statistics Yearbook
SSEZ	1584	78.60	0	5.484	7.294	China Development Zone Review Announcement Catalogue (2018)
*GS*	1584	607.0	0.000424	12.32	39.86	MRIO table [42]
*gdp*	1584	50.63	0.618	5.316	5.078	China City Statistical Yearbook
*structure*	1584	89.30	14.90	49.45	10.11	China City Statistical Yearbook
*tech_exp*	1584	1.048	0.000627	0.0176	0.0433	China City Statistical Yearbook
*open*	1584	1.876	0	0.283	0.276	China City Statistical Yearbook
*consume*	1584	14.58	0.0481	1.875	1.697	China City Statistical Yearbook
*population*	1584	26.48	0.0509	4.413	3.421	China City Statistical Yearbook

Source: Own creation, results of Stata (StataCorp, College Station, TX, USA).

**Table 2:** Baseline regression results (Model 1).

Variables	(1)	(2)	(3)	(4)	(5)	(6)	(7)
SSEZ	−1.003 ***	−0.953 ***	−0.921 ***	−0.937 ***	−0.938 ***	−0.914 ***	−0.882 **
	(−2.80)	(−2.72)	(−2.70)	(−2.76)	(−2.76)	(−2.66)	(−2.52)
*gdp*		−0.302	−0.488 *	−0.344	−0.344	−0.148	−0.182
		(−1.17)	(−1.81)	(−1.22)	(−1.22)	(−0.45)	(−0.55)
*structure*			0.120 ***	0.112 ***	0.113 ***	0.095 ***	0.080 ***
			(4.64)	(4.35)	(4.28)	(2.99)	(2.67)
*tech_exp*				−11.315 ***	−11.293 ***	−11.885 ***	−29.918 ***
				(−2.72)	(−2.71)	(−2.87)	(−3.40)
*open*					−0.099	−0.192	0.049
					(−0.25)	(−0.50)	(0.12)
*consume*						−0.569	−0.360
						(−1.32)	(−0.77)
*population*							3.608 ***
							(2.88)
*Constant*	38.447 ***	39.778 ***	34.662 ***	34.566 ***	34.572 ***	35.387 ***	20.049 ***
	(19.51)	(16.12)	(15.14)	(15.12)	(15.08)	(14.84)	(3.05)
*Obs*	1584	1584	1584	1584	1584	1584	1584
*N*	264	264	264	264	264	264	264
*Adj−* $R^{2}$	0.996	0.996	0.996	0.996	0.996	0.996	0.996
*City FE*	YES	YES	YES	YES	YES	YES	YES
*Year FE*	YES	YES	YES	YES	YES	YES	YES

Note: Robust *t*-statistics in parentheses; *, **, *** indicate *p*-value < 10%, 5%, and 1%, respectively. Source: Own creation, regression results of Stata (StataCorp, College Station, TX, USA).

**Table 3:** DID regression results (Model 4).

Variables	(1)	(2)	(3)
*D*	−175.390 **	−152.985 **	
	(−2.50)	(−2.11)	
$D^{-2}$			−129.875
			(−1.10)
$D^{-1}$			−132.027
			(−1.03)
$D^{0}$			−248.595 **
			(−2.22)
$D^{1}$			−172.852 *
			(−1.69)
$D^{2}$			−154.308 *
			(−1.85)
*Constant*	8387.736 ***	12,356.931 ***	8545.559 ***
	(239.20)	(8.81)	(168.09)
*Obs*	1566	1566	522
*N*	261	261	261
*Adj−* $R^{2}$	0.987	0.988	0.0256
*Control*	NO	YES	YES
*City FE*	YES	YES	YES
*Year FE*	YES	YES	YES

Note: Robust *t*-statistics in parentheses; *, **, *** indicate *p*-value < 10%, 5%, and 1%, respectively. Source: Own creation, regression results of Stata (StataCorp, College Station, TX, USA).

**Table 4:** Mechanism examination results.

	(1)	(2)	(3)	(4)
Variables	*GS*	*GS*	*Emission*	*Emission*
SSEZ	5.083 ***	3.459 ***	−0.751 ***	−0.718 **
	(3.82)	(3.04)	(−2.62)	(−2.53)
*GS*			−0.049 ***	−0.056 ***
			(−4.31)	(−3.55)
*Constant*	−15.555 **	−37.213 ***	37.678 ***	16.731 **
	(−2.13)	(−3.64)	(22.90)	(2.57)
*Obs*	1584	1584	1584	1584
*N*	264	264	264	264
*Adj-* $R^{2}$	0.903	0.930	0.996	0.996
*Control*	NO	YES	NO	YES
*City FE*	YES	YES	YES	YES
*Year FE*	YES	YES	YES	YES

Note: Robust *t*-statistics in parentheses; **, *** indicate *p*-value < 5% and 1%, respectively. Source: Own creation, regression results of Stata (StataCorp, College Station, TX, USA).

**Table 5:** Results of robustness check.

	(1) Exclude the Sample of Cities without New Zones			(2) Change the Indicator for SSEZ			(3) Change the Indicator for *GS*		
Variables	*Emission*	*GS*	*Emission*	*Emission*	*GS*	*Emission*	*Emission*	*GS*	*Emission*
SSEZ	−1.003 ***	4.007 ***	−0.767 ***	−0.886 **	3.458 ***	−0.721 **	−0.882 **	1.961 **	−0.768 ***
	(−2.72)	(3.57)	(−2.86)	(−2.53)	(3.02)	(−2.54)	(−2.52)	(2.35)	(−2.59)
*GS*			−0.066 ***			−0.057 ***			−0.068 ***
			(−2.75)			(−3.56)			(−3.19)
*Constant*	20.961 ***	−35.458 ***	17.682 **	16.196 **	−22.425 **	13.559 **	20.049 ***	−33.162 ***	17.043 ***
	(2.92)	(−3.50)	(2.50)	(2.56)	(−2.47)	(2.13)	(3.05)	(−4.51)	(2.63)
*Obs*	1182	1182	1182	1566	1566	1566	1584	1584	1584
*N*	197	197	197	261	261	261	264	264	264
*Adj-* $R^{2}$	0.997	0.935	0.997	0.996	0.930	0.996	0.996	0.917	0.996
*Control*	YES	YES	YES	YES	YES	YES	YES	YES	YES
*City FE*	YES	YES	YES	YES	YES	YES	YES	YES	YES
*Year FE*	YES	YES	YES	YES	YES	YES	YES	YES	YES

Note: Robust *t*-statistics in parentheses; **, *** indicate *p*-value < 5% and 1%, respectively. Source: Own creation, regression results of Stata (StataCorp, College Station, TX, USA).

**Table 6:** Endogeneity test.

	(1)	(2)	(3)	(4)	(5)
	First Stage	First Stage	Second Stage	Second Stage	Second Stage
Variables	${\mathrm{SPZ}}_{\mathrm{size}}$	*GS*	*Emission*	*GS*	*Emission*
*I_rdls × 2011*	−0.005 ***				
	(−6.03)				
*I_rdls × 2012*	−0.003 ***				
	(−3.63)				
*I_rdls × 2013*	−0.001				
	(−1.00)				
*I_rdls × 2014*	−0.001				
	(−1.41)				
*I_rdls × 2015*	−0.001				
	(−0.67)				
${\mathrm{GS}}_{t-1}$		1.022 ***			
		(162.35)			
SDZ			−5.181 ***	11.168 **	−4.539 ***
			(−4.22)	(2.56)	(−3.77)
GS					−0.055 ***
					(−5.09)
*Obs*	1584	1320	1320	1320	1320
*N*	264	264	264	264	264
*F-statistics*	10.18	5348.76			
*Control*	YES	YES	YES	YES	YES
*City FE*	YES	YES	YES	YES	YES
*Year FE*	YES	YES	YES	YES	YES

Note: Robust *t*-statistics in parentheses; **, *** indicate *p*-value < 5%, and 1%, respectively. Source: Own creation, regression results of Stata (StataCorp, College Station, TX, USA).

**Table 7:** Heterogeneity analysis by industry structure grouping.

	(1)	(2)	(3)	(4)	(5)	(6)
	Low-Ratio Group			High-Ratio Group		
Variables	*Emission*	*GS*	*Emission*	*Emission*	*GS*	*Emission*
SSEZ	−0.035	0.515 **	0.058	−1.323 ***	4.404 ***	−1.144 ***
	(−0.36)	(2.54)	(0.45)	(−2.80)	(2.89)	(−2.79)
GS			−0.209 *			−0.044 ***
			(−1.93)			(−3.42)
*Constant*	−1.194	−4.408 *	−3.353	34.007 ***	−57.654 ***	30.715 ***
	(−0.12)	(−1.70)	(−0.32)	(4.90)	(−2.73)	(4.44)
*Obs*	780	780	780	765	765	765
*N*	169	169	169	174	174	174
*Adj-* $R^{2}$	0.995	0.899	0.995	0.996	0.931	0.997
*Control*	YES	YES	YES	YES	YES	YES
*City FE*	YES	YES	YES	YES	YES	YES
*Year FE*	YES	YES	YES	YES	YES	YES

Note: Robust *t*-statistics in parentheses; *, **, *** indicate *p*-value < 10%, 5%, and 1%, respectively. Source: Own creation, regression results of Stata (StataCorp, College Station, TX, USA).

**Table 8:** Heterogeneity analysis by green technology stock grouping.

	(1)	(2)	(3)	(4)	(5)	(6)
	Low Green Technology Stock Group			High Green Technology Stock Group		
Variables	*Emission*	*GS*	*Emission*	*Emission*	*GS*	*Emission*
SSEZ	−0.127	0.694 ***	−0.025	−1.187 ***	3.812 ***	−1.038 ***
	(−1.10)	(2.79)	(−0.20)	(−2.77)	(2.72)	(−2.82)
GS			−0.159 ***			−0.043 ***
			(−2.59)			(−3.35)
*Constant*	−15.281	−3.261	−16.967 *	46.813 ***	−69.387 ***	43.287 ***
	(−1.49)	(−1.36)	(−1.66)	(7.63)	(−3.35)	(7.36)
*Obs*	860	860	860	715	715	715
*N*	167	167	167	147	147	147
*Adj-* $R^{2}$	0.994	0.771	0.994	0.997	0.932	0.997
*Control*	YES	YES	YES	YES	YES	YES
*City FE*	YES	YES	YES	YES	YES	YES
*Year FE*	YES	YES	YES	YES	YES	YES

Note: Robust *t*-statistics in parentheses; *, *** indicate *p*-value < 10% and 1%, respectively. Source: Own creation, regression results of Stata (StataCorp, College Station, TX, USA).

**Table 9:** Heterogeneity analysis by administration hierarchy.

	(1)	(2)	(3)	(4)	(5)	(6)
	National Zones			Provincial Zones		
Variables	*Emission*	*GS*	*Emission*	*Emission*	*GS*	*Emission*
SSEZ	−1.904 ***	7.057 ***	−1.589 ***	−0.360	1.723	−0.282
	(−2.85)	(4.19)	(−2.73)	(−1.01)	(1.24)	(−0.94)
GS			−0.051 ***			−0.063 ***
			(−3.77)			(−3.19)
*Constant*	18.242 ***	−30.601 ***	15.529 **	16.903 ***	−24.024 **	13.789 **
	(2.84)	(−3.45)	(2.39)	(2.60)	(−2.33)	(2.13)
*Obs*	1566	1566	1566	1566	1566	1566
*N*	261	261	261	261	261	261
*Adj-* $R^{2}$	0.996	0.932	0.997	0.996	0.927	0.996
*Control*	YES	YES	YES	YES	YES	YES
*City FE*	YES	YES	YES	YES	YES	YES
*Year FE*	YES	YES	YES	YES	YES	YES

Note: Robust *t*-statistics in parentheses; **, *** indicate *p*-value < 5% and 1%, respectively. Source: Own creation, regression results of Stata (StataCorp, College Station, TX, USA).

## Data Availability

The data that support the findings of this study are available on request from the corresponding author. The data are not publicly available due to privacy or ethical restrictions.
